# “Making a difference”: Interpreting responsivity ambience for parole work

**DOI:** 10.1016/j.heliyon.2024.e39617

**Published:** 2024-10-19

**Authors:** Micheal P. Taylor, Rosemary Ricciardelli, Randy Shively

**Affiliations:** a Ocean and Public Safety Laboratory, Memorial University of Newfoundland's Fisheries and Marine Institute, 155 Ridge Road, St John's, NL, A1C 5R3, Canada; b Alvis, 2100 Stella Court, Columbus, OH, 43215, USA

## Abstract

In this article, we draw on qualitative interview data (n = 150) from parole officers (POs) employed in Canada's federal correctional service. Our analysis interprets job satisfaction, accountability, and relational aspects of POs' work, taking a semi-grounded constructivist approach. We discovered correctional workers, such as probation and parole officers, engage in transformational relationships within their workplace environments. However, given economic, social, and political constraints, we question how these change agents actually ‘make a difference’ in practice. Fidelity to core correctional practices suggests therapeutic alliances are fundamental to intervention. Emergent in our discoveries is how the workplace environment, organizational climate and culture, and penal atmosphere mediate reflexive experiences that inspire motivation, morale, and change. However, our interpretation and situational awareness of parole adds to a lacuna in knowledge about therapeutic relationships in correctional work generally and the responsivity principle specifically for sensemaking about how interventions may sometimes become iatrogenic. We found that POs negotiate their relationships with those under supervision as much as with fellow correctional workers. *Responsivity ambience* pertains to consolidation and conceptual clarity concerning how corrections, in its public safety mandate, induces in/efficacy. Through discussion, we theorize how securitized settings affect well-being and provide practical insights to converge on the rehabilitative ideal and criminal desistance.

## Highlights

• Elucidates situational awareness on parole/probation as public safety work.
• Explores a multilayered perspective on socio-political economy in criminal justice.
• Finds a need to further study ideological commitments in penal practice.
• Inspires novel interpretations of operational efficacy and ‘responsivity’ principle.
• Creates understanding for systems thinking about relationships in criminalization.

## Graphical abstract

**Figure:**
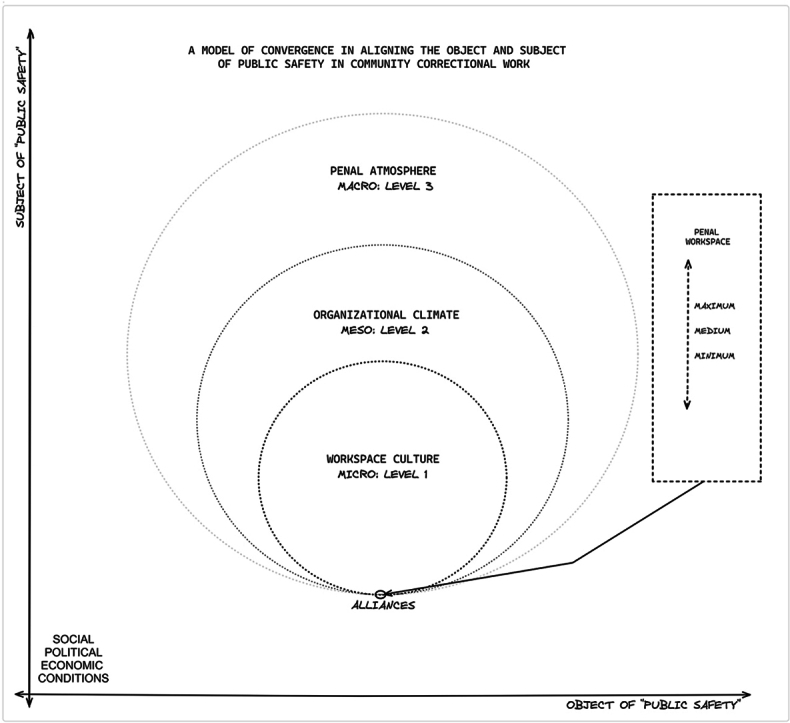

## 1 Introduction

In this article, we interpret findings from a Canadian study on parole work—the equivalent of probation in some jurisdictions—which occurs in prison and community settings. We explore our findings on the enterprise of public safety work through a lens of situational awareness about correctional intervention. In doing so, we elucidate micro, meso, and macro levels of interpretation and operationalize awareness about the psychosocial hazards associated with parole workplace environments [[1], [2], [3]]. Specifically, we draw on social psychology and penological theory [[4], [5], [6], [7]], to elucidate correctional work in terms of operational efficacy also known as the responsivity principle in practice [8,9]. We show how parole officers (POs), operating in carceral workspaces that traverse prison and community settings, deserve recognition for their contribution to public safety [[10], [11], [12]]. In doing so, we present parole in terms of community correctional work which mediates social, political, and economic in/security.

We begin with a thematic literature review about the parole situation in Canada and the responsivity principle in theory and practice. Then, we discuss our methodological approach to data collection and analysis. In our results, we explore various levels of interpretations traversing micro/meso perspectives in the context of alliances. We frame our findings about individual casework with clients (micro level), sometimes referred to as a “therapeutic alliance” as mediated by “working alliances,” otherwise referred to as teamwork among staff (meso level). In our discussion, we elaborate on the macro-level perspective of our findings, highlighting some theoretical and practical implications. We then conclude with a concise summary of how community correctional work might better align with the rehabilitative ideal using the responsivity principle [13]. Thus, we ask what it means to “make a difference” for parole officers working as change agents and how socio-economic and political factors impact their public safety work.

Our findings draw from qualitative data from a study by Ricciardelli et al. [14] investigating the conditions of federal POs' (n = 150) organizational climate and culture within Correctional Service of Canada (CSC). Federal parole in Canada is distinct from other supervisory programs, such as probation. While our focus derives from an awareness that POs' workload demands have, to date, received limited scholarly attention in Canada despite a national strategy for investigating operational stress injuries among public safety personnel [11]. We recognize that probation and parole officers’ roles overlap to varying degrees in Canada and thus use PPOs to demonstrate their shared reality [15]. However, we distinguish federal POs when necessary to denote data collection drawn from Ricciardelli et al. [14], which was a study commissioned by the Union of Safety and Justice Employees (USJE) [10].

## 2 Literature review

Probation and parole officers (PPOs) engage in public safety work by supervising and assisting criminalized people—pre and post-incarceration—within prisons and community settings, creating a complex situation that traverses various levels of awareness [2,16]. On a micro level, PPOs *operate* individually at their local workplaces doing casework [17]. On a meso level, PPOs collectively engage in teamwork with other varieties of correctional workers, altogether exercising *judgment* within a penal organization [[18], [19], [20]]*.* Hence, on a macro level, PPOs represent public *confidence* in the criminal justice system in their knowledge translation and action while negotiating research, policy, and practice [21]. In Canada, probation officers are employed largely at the provincial/territorial level and parole officers (POs) tend to work at the federal level, nationally, supervising adults incarcerated for a prison term of two years or more [22,23]. As in other jurisdictions, Canadian PPOs supervise criminalized people, per judicial orders, where they increasingly report experiencing ambiguity and role conflict around their managerial function conducting risk assessments [18,19,[23], [24], [25]]. In other words, uncertainty is a pervasive feature of Canadian probation and parole work, associated with occupational stressors including symptoms of anxiety, depression, and posttraumatic stress [[26], [27], [28]].

Despite experiencing compromised mental health, PPOs play a pivotal role in public safety within Canada's penal system [1,10,11,13]. Approximately 1300 POs are employed federally by the Correctional Service of Canada (CSC), operating within prison and community workplaces [29,30]. Their clientele consists mostly of prison residents (n = 13,900), where institutional parole officers (IPOs) prepare prisoners for release and community parole officers (CPOs) support the reentry of “offenders” (n = 9100) into the community according to parole conditions. Therefore, IPOs and CPOs operate in distinct workplace environments. However, despite their unique functions, collectively, they converge on rehabilitation to support criminal desistance [13], and their roles and responsibilities are informed by their respective workplace culture [29]. In line with Yurtsever and de Rivera [31], Luo [1] and others, we distinguish climate and culture for this article as environmental units about the penal atmosphere [32,33]. Climate refers to how individuals perceive and relate knowledge about their environment, whereas culture pertains to the actions, values, and behaviours imbued through people's relationships [21,31]. Workplace environment distinctions are specific, while organizational climate and culture are diffuse. These micro/meso-level perspectives explicate a macro-level dimension regarding the overall milieu in which penal systems are situated and operate given social, economic, and political imperatives [13].

The *responsivity principle* forms the lesser-known component of the most prominent correctional intervention framework globally—sometimes called the Risk, Need, and Responsivity (RNR) model—and refers to person-intervention fitness [34]. In brief, the risk principle refers to *who* needs interventions, the need principle is about *what* needs to be intervened, and the responsivity principle is about *how* to intervene. While the risk—and, to a lesser extent, need—principles have been well established through decades of meta-analytical research and theoretical refinement, the responsivity principle remains relatively underdeveloped. For example, despite responsivity constituting a core principle of correctional practice [35], it is generally studied in community-based rather than institutional correctional settings [9]. Also, several dimensions exist in the literature (i.e., internal, external, general, specific, systemic, and political), which all tend to relate from the micro-level perspective of service-user engagement [[4], [5], [6],^9^]. However, more recently, responsivity has been conceived regarding practitioners’ needs [5]. The responsivity principle requires theoretical integration to form more explicit implications in practice.

Moreover, “therapeutic alliance” and “working alliance” are often referenced indistinguishably in the literature about casework among clients/practitioners [8,36,37]. However, our findings suggest the need to differentiate the former from the latter, where working alliances connote an understanding of teamwork and staff cohesion [38]. A common characteristic all correctional workers share is their vocation to human service, and PPOs are particularly recognized for embracing this ideological commitment [15,16]. However, burnout is a human cost related to the various psychosocial hazards of correctional work and its tolling nature [3,16,18,19,23,[26], [27], [28],^39^]; especially given the professional exchange of pay for emotional labour which otherwise may be reserved for kin or persons related to the practitioner [40]. In the present article, we explore micro-level and meso-level interpretations of “correctional therapeutic relationships” [41] while informing the lacuna of knowledge about correctional workers’ teamwork and, in doing so, advance knowledge about how bonding, task agreement, and building mutual consensus occurs from the ground up [42,[43], [44], [45]].

### 2.1 Level 1: micro perceptions of awareness

On a micro level, the enterprise of community correctional work occurs on a case-by-case basis [1]. Likewise, as individuals, PPOs enter the field because of their interest in human service and working in relationship with people, arriving at their role with various personality traits [16]. In line with research on correctional officers [[46], [47], [48]], it is theorized that PPOs are motivated by their orientation and perceptual awareness of clients’ needs [17,37,49]. Penological theory holds that “specific responsivity” refers to tailoring interventions with sufficient specificity as to align practitioners/clients [7,35]. Endsley [2] poses that the quality and sufficiency of information to form judgements at this level is premised on the ambiguity or reliability of individual perceptions. Therefore, PPOs experience their perceptual understanding as mediated by the quality of their workplace relationships [16,37,50,51].

### 2.2 Level 2: meso interpretations of meaning and comprehension

On a meso level of interpretation, workplace characteristics “fit” within organizational contexts and are situationally interpreted based on the unique traits PPOs bring to their role [52,53]. As Worrall and Mawby [16] observed while investigating PPOs' occupational culture in the United Kingdom, pivotal to probation and parole is the “belief in people to change.” Thus, PPOs are ideologically committed to the rehabilitative ideal [13]. Through socialization, a meso-level interpretation suggests that personalities amalgamated with workplace characteristics intermix in a matrix of workspace functions and across the varieties of work, as differentiated by IPO and CPO roles [20]. Thus, IPOs, CPOs—and PPOs broadly conceived—represent a formative group of socially networked relationships within an organizational climate [1,31]. Hence, the “general responsivity” principle means correctional workers aim collectively to foster a learning environment that optimizes all people's needs [[4], [5], [6],^54^].

However, Gayman and Bradley [39] found in practice a sample of PPOs (n = 825) working for a North Carolina Department of Corrections experience work stress and depression, which undermines the capacity and impact of general and specific responsivity. The researchers concluded the association between the organizational climate and mental health may be “spurious” (p. 339). Nevertheless, PPOs were observed there, as elsewhere, to experience ambiguity and role conflict, suggesting a relationship between the environment and issues about workload, burnout, and staff turnover [[55], [56], [57], [58]]. According to Endsley [2], governance at the meso-level pertains to an awareness of mental models shaped by environmental factors. Hence, “systemic responsivity” implies issues with the climate or culture of operations [6]. These represent an amalgamation of socially imbued workplace units and inter/intra governmental departments that provide context for “internal” and “external” responsivity when investigating penal organizational phenomena [1,9,33].

### 2.3 Level 3: societal projections and macro implications of “public safety”

At the macro level, a metacognitive awareness emerges about institutional bias and processes that may induce or reduce criminalization [59], which might be conceived generally as “political responsivity” [54]. Here, personality differences are socialized anew through projections related to workplace characteristics internally within the organization [52,53]. According to Endsley (2020), these variables are explained as a divergence between object and subject. For instance, as Luo (2020) observes, one possible consequence is the enculturation of ideological commitments through socialization, where in-group correctional workers influence out-groups [38]. As such, systemic and political responsivity considered suggests penal practitioners may not fully recognize how their behaviour affects others and may render them overconfident despite being cognitively unaware of their actions’ implications [2,21].

An illustrative example may be how members of an organization experience their work (e.g., internal responsivity) and subsequently inform projections about their organization's image or reputation (e.g., external responsivity) which has implications for public confidence. Evidence of internal responsivity issues in parole workplaces includes harassment and “spiralling incivility” [3,60]. Therefore, the object of rehabilitation diverges when subjective experiences demonstrate widespread hostility or negativity bias [61,62], undermining program integrity which compromises fidelity to core correctional practices. At best, operations are rendered ineffective, or worse, the degree to which interventions become unresponsive can also present hazards so that correctional work turns iatrogenic [63,64]. Hence, a macro-level interpretation is that responsivity is an adaptation to the ambient penal atmosphere.

## 3 Methods

Approval for Ricciardelli et al. [14] was granted by the Research Ethics Board at Memorial University of Newfoundland and a fulsome account of this project has been reported eslewhere [29,30]. In brief, recruitment materials were disseminated by CSC and USJE via internal listservs containing information with instruction on how to contact the principle investigator for those who wished to participate. Informed consent was obtained prior to participation including agreement to audio-recorded telephone-based interviews. Due to geographical limitations, resource constraints, and public health measures coinciding with a global pandemic when data was collected, all interviews were conducted by phone. Most interviews lasted between 75 and 120 min and all occurred in August–September 2020 [14,29]. We found telephone-based interviews effective in establishing rapport and aligned with research validating the phone as a reliable mode of in-depth qualitative interviewing [[65], [66], [67], [68], [69], [70]]. In fact, we would argue that a lack of eye contact enables “disinhibition” for participants making it easier to facilitate disclosure [71,72], when discussing sensitive topics such as mental health and employment consternation. An informal snowball sample supported our formalized recruitment strategy which meant coworkers referred colleagues on their own accord.

In practice, interviewers posed broad questions to participants enabling POs to share what they felt was most relevant followed by probing for clarification [73]. We took to research using a semi-grounded constructivist approach [[74], [75], [76], [77]], meaning we allowed themes to emerge iteratively. In brief, grounded theory is about inductive research that produces richness through descriptive accounts, through narrative analysis, based on criterion, namely, a high degree of specificity when reporting on findings [78]. While a limitation of this approach is its lack of generalizability in other research domains, as an interpretive approach it creates an opportunity to reflect on insights which may otherwise go unnoticed [79]. In other words, grounded-theory is “constructivist” because, as researchers, we recognize how our prior understandings position how we investigate the field and use reflexivity as a technique to situate our analysis [19,80].

Criteria for assessing our semi-grounded constructivist approach means considering our discoveries in terms of credibility, originality, resonance, and usefulness that build upon the research traditions of pragmatism and symbolic interactionism [74,79]. Therefore, we refrained from imposing theoretical presumptions, despite recognizing the significance that our respective exposure to this field of inquiry bring to analysis [19,81]. Rather, we privileged PO participants' experience regarding whatever arose thematically across all interview data. Hence, we remained grounded to POs' interpretations while also recognizing that our analysis represents a constructed mix of their and our interpretations. In practice, this means interviewers probed about insights which were, in part, gleaned from prior studies and the materializing of such data form the basis of emergent discoveries [82,83]. Generally, those discoveries were guided by interviewers’ loose adherence to a topic list which featured questions about job satisfaction, health, and the well-being of participants in light of them working during COVID-19 [14].

Interview data was coded, inductively, first by three members of our research team sequentially ‘open coding’ five interviews, independently, based on what themes resonated with the greatest salience [74,78]. Once an initial set of codes was established from that preliminary exercise, the remaining transcripts were axially coded, meaning, as new observations arose the team would reconcile discoveries through discussion and revision [78]. Finally, a codebook emerged through iteration. This process provided a qualitative variant of inter-rater reliability, meaning, we triangulated our observations across investigators until consensus was met [84,85]. QSR Nvivo software aided coders but no auto generative features were employed. Axial coding of all transcript data (n = 150) resulted in a codebook featuring ∼130 discernible themes. The present findings are based on an analysis of three of those themes: *job satisfaction* was interpreted as POs' descriptions of what was enjoyable in their work. *Relational aspects* denoted the qualities POs' observed in their interactions with parolees, colleagues, and others. And, *accountability,* was a theme related to the varieties of roles and responsibilities collectively necessary in representing the team effort for realizing rehabilitation and criminal desistance.

In a final iteration, the first author coded selectively to develop a sequence of categories and construct a coherent analytical narrative of all discoveries reported in this article [78]. In practice, selections meant data was tested conceptually against the tenets of constructivist grounded theory [74]. The valuation for discarding unreliable interpretations accorded to credibility, originality, resonance, and usefulness critera and interpretations we present are descriptive excerpts of transcript analysis in our findings.

### 3.1 Participants

We limit further discussion to the present findings and showcase demographic data for participants (see Tables 1, 1.1, 1.2, and 1.3) based on those who emerged from our thematic analysis. Of note, some participants had transitioned into managerial or supervisory roles at the time they were interviewed. However, all participants (n = 30) reflected on their job tenure as a PO. We found most participants were female (n = 25), between the age range of 35–44 years (n = 17), and predominantly IPOs (n = 18) working within minimum, medium, and maximum security prison settings. The remaining PO participants worked within the community, as CPOs (n = 12). The IPO/CPO designation provides an understanding about where the participant predominantly worked during their PO tenure [14].

Collectively, POs brought experiences, ranging from employment within provincial probation and victim services, policing, and child welfare work. Our findings consist of excerpts from transcript data which have, at times, been revised for confidentiality, grammar, and syntax. However, we always remain accurate to the verbiage and meaning posed by participants during interviews. In our results, we present anonymized data by detailing participants' identification number (PIN) assigned when their data was collected and circumvent bias inherent to pseudonyms. Assigned designations for IPO or CPO are also presented along with an indication of the few male participants (i.e., ‘M’; n = 4). One participant elected to not identify their gender.

## 4 Results

We found IPOs and CPOs collectively enjoyed most “working directly with offenders” (CPO24, CPO54). For instance, CPO39 explained, “seeing the guys on my caseload, talking to them about their life, what they're doing for work, their interests, their leisure activities” was central to parole work. POs described most valuing the human service element for learning about and working closely with their clientele. Moreover, working with people extended beyond dealing with service users and involved also working in a professional environment. IPO112 (M) said, “I like being able to sort of work with all these different people and different even personalities … there's quite of bit of socialization”. POs valued most working with people, including both service users (i.e., clientele) and service providers (i.e., colleagues). As IPO20 stated, “the part I enjoy most is just having conversations with people and like. … interaction with people … inmates and staff”. Therefore, responsivity was evident in how POs described valuing their relationships at work. In the following sections, we unpack these experiences in terms of micro and meso levels of abstraction, emphasizing the working and therapeutic alliances amongst parolees and colleagues that POs describe in their collaborative effort for rehabilitation.

### 4.1 The micro: bonding through care work

We’re supposed to be interacting and positively role modeling so when they’re returned into the community they’re not more bitter and twisted … You know, [then] when they came in—and [I’m] not saying that there’s [not] a small percentage of inmates [who] are just anti-social to the core—but ninety-nine percent of the inmates I’ve interacted with over [two decades]—if you’re respectful to them and engage them like adults in an open manner, that pays dividends (IPO2, M).

With these words, POs observed most criminalized people respond well to engagement with care ethics—notwithstanding the few cases of psychopathy or sociopathy where anti-social behaviour is believed to be impervious to change [86]. Inspiring change requires being open to interpersonal growth. Caring, for POs, meant engaging people meaningfully based on their unique circumstances. As stated, “there must be something good about parole, there must be something extra, I don't want to say kindness but something [so that] they're willing to go a little bit further” (IP064, M). Service users need to be shown care and respect. For POs, engaging clients meant both a desire to inspire and informatively, also the ability to become inspired by clientele:

My favourite part of the job is having the privilege of observing other peoples' lives and learning from them. I watch[ed] somebody who had a really difficult life pass away from natural causes and it was kind of a longer process he went through, but he handled it with so much grace and strength and to watch the family [was inspiring]. It was an Indigenous person who really struggled but he maintained his sense of humour … he made the right choices for his health in terms of what he wanted, the decisions he wanted to do in the last few years of his life, and he didn’t have any regrets, and that’s just a small example of how the meaningfulness that you get, and those moments are, they’re not often but they’re very powerful when they’re there. … [I]t’s very rewarding to be a witness to that (IPO27).

These words evidence the “grace” for witnessing a client's death as inspirationally ‘touching’ for the PO [87]. The therapeutic alliance was so meaningful that it fostered a bond enabling the PO to reflect on their own life circumstances. Another stated, “being on the frontline. Doing follow-ups with certain inmates. Learning more about their criminality. … just being there like face-to-face doing those kinds of interviews. … Challenging them, making them realize things” (IPO119) were other sources of inspiration. For them, witnessing growth meant helping to facilitate learning through interpersonal education.

Realizing change occurred through relationships thus served service users and providers alike which meant the alliance was seen to be therapeutic and fostered meaning in life. In this way, CPO18 remarked, “I like meeting different people, learning about their stories and their backgrounds and their goals … I like helping people”. While this participant recognized “public safety” as the ultimate imperative of PO work, they attended foremost to the purpose of engaging with professional curiosity to help facilitate learning [88]. Public safety was therefore secondary and abstract to the tangible knowledge about creating change in client's circumstances. As IPO6 stated, “I think the only reason I have any pride in any of my work is that I know there's like those few and far between cases where like I really get through to a guy and I really believe I've helped him and he's not going to re-offend as a result”. For POs, gratification was gained through knowledge about clientele and the practice of care was an ethic that inspired both teaching and learning. Qualifying this observation was evidenced in the following words:

What I do matters to me, and the people, the offenders matter to me, and obviously … the relationship, the rapport that I have with my offender impacts society … We have a pretty privileged role we can role model and I have a little board up in my office … I would put motivational quotes up there. I would say to my offenders ‘I’m going to hold you to a very high expectation because if I don’t who will? … And I’m going [to] try and help you to reach that—if you want to work with me, if you don’t waste my time—just have the courtesy and the respect to tell me and then I won’t invest time in you and I’ll invest in somebody else who is ready to work with me.’ … And I also tell them ‘I’m not going to work harder then you if you want to work hard, if you’re willing to work hard, I will work equally as hard to help you achieve your goals, but I won't work harder then you’ (IPO109).

As these words indicate, echoing others, the theme of care ethics emerged when discussing the extent to which bonding inspired rehabilitation. Therefore, POs tended to be eager to meet clients and invest time and energy to form the therapeutic alliance and, in doing so, the aim was to inspire interpersonal growth. Thus, POs described ‘working hard’ to form alliances and collaborate in the shared responsibility of teamwork as a core principle and ideological commitment to supporting parolees with re-entry.

#### 4.1.1 Engaging in the bond

POs worked to build relationships and establish rapport by recognizing their client's needs based on whatever stage the client found themselves on their desistance journey. IPO23 recalled once saying to a particular client: “‘you're not a bad guy … we can make some changes' … most of the guys I find, especially when you have an opportunity to have long conversations on a regular basis, it really does *make a difference*”. They explained that demonstrating care meant engaging clients—through collaborative language—by finding the time to converse. They created a sense of value for the parolee for a return on investment in the relationship. To demonstrate the depth of the investment, it was explained:

I like working with guys and that’s one of the reasons I wasn’t too keen on being a supervisor. If I were to ever take a supervisor position I would still wanna keep some guys on my caseload cause I do like working with the guys and once in a while you get feedback from the guys when they’re done, and they’re really happy to have met you (CPO107).

With these words, value in casework was described when clients reciprocated appreciation and demonstrated a return on POs' investment. POs contributed to the parolee's stability by investing their time and creating the space necessary to form a bond—i.e., “keep guys on the caseload.” Thus, relationships created a sense of purpose and meaning for POs doing parole work. Working *with* clients meant casework brought the most satisfaction and was enjoyable:

I really do like working with the inmates like believe it or not (laughter) some days I want to pull my hair out, but I really do like engaging with them and I find that I have a really good rapport with a lot of them if they’re willing … I really do like engaging with the guys (IPO139).

These words demonstrate that relational work with clients was most valued by POs and engaging in therapeutic alliances was responsive collectively to PO and client needs alike. Another PO described a similar observation:

… interviews directly with inmates are the most interesting thing for me … we can read the file for years, but meeting the person is completely different. … it’s not something that is considered very important for correctional services. There’s a lot of emphasis put on the legal side and the paperwork side … but those contacts are important, and I think that for the inmates as well that more human contact is very important in their lives and in their eventual reintegration (IPO146).

Emergently, POs' narratives underscored the value placed on relationships where “human contact” with the clients was pivotal to their role. However, emerging from this narrative were the challenges that undermined POs' capacity given certain administrative burdens, such as paperwork, a necessity for maintaining legal compliance, but also, evidencing a divergence between rehabilitative work and time constraints or other conflicting pressures which devalued what POs described as most meaningful.

#### 4.1.2 Interpersonal growth and witnessing a shift

Crafting change management for each unique case was a challenge POs had to negotiate in their supervisory practice by situating their work as valuable and crafting responses to client's needs. As IPO141 described, “the one-on-one with the offenders … the impression that you can help, that you can give good advice, and you see like the change in the person” was inspiring. Thus, meaning was attributed to the relationships that were most responsive to shifting perspectives.

I feel like I can—for all of the tragedy and damage and sorrow and pain that I’ve read … thankfully—I have at least, I feel, *made a difference* in a few peoples' lives. I’m glad I came to work on a few days cause I really feel like I did *make a difference* (IPO109).

With these words, POs show up to “make a difference” through their humanistic relationships, namely, developing a therapeutic alliance with clients. Occupationally, then, POs’ interactions meant helping to move clients, when incarcerated, into lower tiers of risk/security (i.e., from maximum to minimum) or within the community toward societal reentry:

being able to have an impact, seeing a guy come out of the institution with basically nothing or working and going to school and [setting] like a real realistic life plan and working towards it. You feel confident in the end they’ve passed this stage right. … So I think that’s seeing somebody grow I guess (CPO107).

Growth here is evidenced when seeing clients work toward pro-social lives that in turn contributes to society. Negotiating such life circumstances, and parole conditions, meant traversing prison and community space. Ideally, by moving from most to least restrictive (i.e., maximum security/intensive supervision) to a space where personal autonomy could be exercised fully in realizing one's betterment. Instances of that shift were described as noticeable movements toward growth:

… being able to make change … I have seen people change. I’m in the process right now of writing a reference letter for one of the offenders on my caseload who’s asking to have his tattoos removed and if he can [have] a character reference on him—he would be able to get free tattoo removal and that’s in order for him to distance himself from his gang network and all of that … so for me to be able to write that letter to help him, which will eventually help with his reintegration, like that makes me feel great (CPO132).

hands on work with the guys on the caseload, like you’re actually *making a difference* … because it is the last stop before they’re out the community—and the ability to make that recommendation … we spend so much time with them … you have a really good understanding (IPO131).

Growth, as indicated in the first excerpt, was observed by the PO's realization about the therapeutic alliance through which the client sought advocacy. By seeking assistance, the prison resident demonstrated their agency, their readiness to move away from their old lifestyle. In the latter example, the PO described emphatic knowledge about their clients' needs, a bond in which they described the “last stop” before moving from prison to society. These words evidence how “making a difference” leads to growth. Thus, representing sources of education and inspiration which were rewarding: “what I like[d] most was the contact with … the guys and doing follow-up … going through the changes with them … and learning … the change process and seeing them change” (IPO147).

### 4.2 The macro: fostering a therapeutic milieu

While investments in time and energy were necessary to form bonds to move clients and inspire life courses anew, the climate for investing in relationships required a rehabilitative focus. Thus, an antecedent to the therapeutic alliance was the occupational environment—the organizational climate—where parole occurs. POs described being stymied by the ‘pains of their employment’ [89], thus, the perception that managers were unfair and unsupportive, tied to resource constraints, meant staffing and other structural issues complicated workplace dynamics and in response hindered working alliances. Nevertheless, POs described valuing most their relationships with people:

I find a lot of value in working with my coworkers. I’ve only been here for under a year, so I don’t really have the network that I had before I came here … it’ll just take time to build that up here, but I felt very valued with the role that I [have], as part of an interdisciplinary team (IPO109).

Satisfaction was derived from teamwork and such working alliances referred to collaborating toward a shared vision. Said differently, if staff feel supported in their environment, alliances between POs and clients and among each other, were innate. To illustrate, CPO57 reflected on their tenure as a PO, before taking on a managerial role:

I like providing guidance to the parole officers too, [for] different approaches, different ideas. Sometimes they’re too involved directly. So when they have an outside view and it gives them the opportunity to say, ‘Okay. I didn’t think of that. Yeah, sure. We should try that.’ I really just like managing people actually. I think I’m kind of good at it.

These words illustrate micro and meso levels of interpretation when POs move from casework to managerial positions and provide guidance to staff. Sometimes, working at the micro level required a need to take a step back when working ‘too closely’ with parolees. Thus, perspectives across roles enhance objectivity. The working alliance and cohesion among staff functioned as a support network for optimizing the correctional environment and promoting a rehabilitative milieu. Vital to human service and change management was the capacity to remain flexible, creative, even playful while locating what was meaningful for clients to purchase [8]. However, a common complaint among POs was how their work was organized, not in terms of human service and relationships, but in terms of paperwork:

Thank goodness I have a psychology degree because that’s the one thing that I think CSC doesn’t account for … they have a bunch of people in programs who don’t have degrees at all. Like [they] have no human service degree background. Like, they may have a grade 12 [high school] and that’s about it. So I don’t know how they process what’s happening there. … I came from that environment and then to come over to parole, I thought I would be doing a little more … one on one stuff. And that didn’t really work out that way. I felt like I was more of like a paper pusher. Like they [clients] would come in and demand that they needed to do this, and this and this and this. And I would just kind of do it for them because they were right, they don’t have access to lawyers or information about furthering their education. So I was finding a lot of information out for people and giving it to them (IPO117).

With these words, the participant described surprise when the normative expectation of using their psychology education in case work with clients was challenged by a focus on administrative processes. Thus, the climate expected “doing” rather than “one on one” relating. What was missing, for this PO, was the ability to inspire growth given their paperwork requirements. They described the environmental challenges in which operations (e.g., referring clients to legal representatives and brokering services beyond CSC) were prioritized over building rapport and engaging in a therapeutic alliance.

#### 4.2.2 Organizational context: space and place

I think having that understanding and getting to know people in a really respectful way is something that’s so needed here. Like our job is risk assessment and ensuring the safety of the public, but we’re also supposed to be supporting rehabilitation. … And I think that can really be bolstered by a positive rapport with the parole officer, and if that’s missing and inmates aren’t feeling heard, or respected, or supported, then, they’re just not going to go as far as they could if they felt like they had that backup and that support (IPO135).

POs, as with the above excerpt, described a duality in their work. On the one hand, they are tasked with administering parole conditions. While on the other, found purpose in facilitating rehabilitation through transformational relationships. By conducting risk assessments and classifying service users into security tiers, the focus was on “public safety”. Whereas tiering clients down meant recognizing opportunities for growth that facilitated the noticeable shift in moving toward desistance through therapeutic alliances. When prisoners felt heard, respected, and supported, personal growth was enabled. However, for such growth to occur POs described the atmosphere as needing to instill the capacity for creating the time and space for relational work to ensue. As such, the correctional climate was often seen as contradictory when POs had to navigate ambiguity and role conflict.

Referring to correctional officers, institutional parole work was sometimes stymied by fellow correctional workers who interfered with and even derailed teamwork:

the CX [correctional officers] group decided that that inmate didn’t belong there anymore—there’s no possibility of reintegrating that person [so] they will be pressuring. We also saw bullying towards some parole officers in some cases where they’re told ‘that makes no sense, don’t do that’ or [CXs] questioning us on our work on the assessments. And sometimes, we don’t feel that we are supported or protected even by management and so maybe less so in medium security than max security, but we can’t go through a gate because they control it. It can add a bit of stress, or we can get some phone calls or some comments [through] emails because … they don’t agree with the assessments that we’ve done, so that can add some stress. … it’s an environment that’s difficult to change so even people who come with good intentions won’t necessarily want to get the CX group against them because they want to keep peace in the workplace. It is culturally set in the organization, but I think the more the level of security increases the worse it is (IPO147).

With these words, the environment emerges as problematic as the conditions for fostering therapeutic alliances are mediated by increases in security classification or the perceived risk posed by the client. When working alliances were compromised by the environment, POs have less ability to bond with clients and colleagues given competing demands. Correctional officers and POs were thus described in tension when institutional security were seen to operate at odds with POs’ capacity and the members were not operating in unison.

I’m currently at … a medium security facility um I’ve done the role of a parole officer at a minimum I’ve done the role of a parole officer at a maximum security … so sometimes there’s different challenges especially as you go higher in security … I just did twelve years at max before I came here so the change has been very good for me … it’s definitely—it’s a more relaxed atmosphere here (IPO12).

Reflected in this excerpt is an environment that reduces autonomy when security management is prioritized over efforts to promote human service. In other words, the climate supersedes the rehabilitative ideal which complicates working alliances among correctional workers.

#### 4.2.3 Professional tenor and collegial relationships

On the other hand, working alliances were more pronounced when a vision was shared about rehabilitative relationships. For instance, POs referred to a high degree of morale when collaboration among staff inspired support for clients:

My favorite part of the job … is the people I work with—I work with great people. My best friend works here so we get to chat and there is a form of camaraderie (IPO23).

I really like the people I work with. … I’ve worked with fantastic coworkers. Both parole officers and correctional officers. I actually enjoy talking to inmates. I find it interesting, their story, where they came from. … COVID is difficult because I’m really limited in having those conversations now (IPO97).

actually helping people has always been my thing. It kind of gives you a good feeling sort of thing but the people I work with, I have to say, [my] coworkers are excellent also … we do for each other … cause I guess we all know what we’re going through (CPO130).

As these POs explain camaraderie made the workplace enjoyable. When job satisfaction was high, it mediated the correctional climate and created capacity for parole work. Central here was how POs enjoyed working with people—both colleagues and clients alike—notwithstanding pandemic conditions that inhibited their work given public health measures. Therefore, what POs valued most was the relationships they maintained in their employment. And particularly salient, given their work represents an insulated society—despite being experienced as tumultuous at times—was the correctional climate which characterized employees' shared knowledge and ideological commitments that sometimes compromised rehabilitation.

## 5 Discussion

Our findings reveal the complexities of therapeutic correctional relationships and conceptual clarity around responsivity ambience across internal, external, general, specific, systemic, and political dimensions. While “alliances” are often presumed in the literature to occur exclusively among service users and providers [36,41,43,44], our findings elucidate shared understandings among staff that suggest differentiation is needed to denote teamwork. As such, we understand working alliances are an antecedent to therapeutic alliances, which, collectively, exist on a matrix of general/specific and systemic/political responsivity dimensions. Therefore, our theoretical contribution provides conceptual clarity regarding occupational stress as evidenced by hostilities experienced in parole workplaces. How do such psychosocial hazards provide for a responsive orientation to bonding, task agreement, and mutual consensus? We argue a lack of conceptual clarity around the responsivity principle can undermine intervention efficacy.

As such, responsivity is ambient, penetrating the workplace culture and organizational climate (i.e., internal responsivity) through people's experiences and relationships within the places where parole exists, regardless of one's role as service user or provider. In turn, such environmental factors imbue parole operations with macro-level understandings (i.e., external responsivity), such as the wholesale reputation of correctional workers or how such public safety work, and its social representations, project low levels of public confidence. As internal/external factors mediate one another, such complexities are vast and highly tied to political rhetoric that informs ideological commitments [5,6] altogether creating divergence from the rehabilitative ideal [13]. According to POs in our sample, relationships were occupationally most valued and denote a need for future research on “care ethics” [90]. In other words, as RNR architects recognize, “the most effective way of decreasing criminal behaviour is to intervene at the human service level” [91], and we agree. However, reordering this model may be necessary to converge on the *object* of criminal desistance and the *subject* of rehabilitation [2].

Therefore, a practical implication of our findings suggests a need to “reconfigure” interventions that orientate policymaking to the unification of all people striving toward well-being [5]. In this sense, responsivity ambience means service users and providers alike exist in relationship to one another, and we posit that job satisfaction and accountability for parole supervision conditions may converge when alignment exists with this relational component [41]. However, we found ruptures in those relationships occurred as POs traversed security tiers and became stymied by the environment while aiming to shift criminalized people from the most coercive to least restrictive settings. If the core of correctional practice is an orientation to human service, we believe workplace tensions mediate the dynamics of space and time where relationships are co-created, negotiated, and constructed [8,37,54]. Thus, practically studying the responsivity principle means understanding how POs change due to their occupation working as change agents [46,47]. We believe an ideological commitment that orients people toward rehabilitation and desistance is core to what it means to “make a difference.”

## 6 Conclusions: limitations and future research

This article unpacks six qualitative dimensions (internal, external, specific, general, systemic, and political) to create conceptual clarity about responsivity ambience in correctional work. Based on interview data with POs, we learned that public safety is observable when changes are seen to “make a difference.” In unpacking those differences across micro/meso levels of interpretation, we note that POs observed a shift in their clients over time by enacting therapeutic alliances. Informatively, we learned that sometimes POs describe their alliance with clients as hindered by operational conditions such as poor human service orientations. Thus, we argue for the need to differentiate working and therapeutic alliances, and in doing so, suggest a unifying principle for correctional workers and service users is the orientation to care ethics. We recognize that systemic issues tied to resource constraints, including social and political pressures make correctional work tolling, especially when socialization over time provokes discord and apathy in a way that turns interventions iatrogenic. Such circumstances are far from a rehabilitative ideal that stymies criminal desistance. Evidence of ill-being are psychosocial hazards associated with correctional work suggests responsivity can induce favourable organizational practices but also reinforce social structures that are potentially criminogenic.

Notwithstanding a novel contribution that consolidates responsivity theorizing, our study is not without limitations. Our analysis is situational and based on a time and place with specific conditions that may not be generalizable elsewhere. Our study design focused on mental health and well-being, shaped our analysis at the preclusion of other avenues of inquiry. For instance, our sample consists of female-identifying participants who, as IPOs, work in a masculinized environment. How do these traits engender nuances depicted in our findings, and in what ways might they provide insight for future research which may be controlled for in the study design phase? Due to the qualitative nature of our inquiry, we cannot isolate variables such as roles and responsibilities to demographic information nor can we discern environmental factors of specific parole workplace units [33]. Altogether, such limitations provide insightful avenues to strengthen future carceral culture and climate research. In doing so, we argue that to ‘make a difference’ means understanding the work longitudinally and would urge researchers to uncover how such views shape public safety conceptions over time, from the perspective of change agents, throughout their career [92].

## CRediT authorship contribution statement

**Micheal P. Taylor:** Writing – review & editing, Writing – original draft, Formal analysis, Conceptualization. **Rosemary Ricciardelli:** Writing – review & editing, Writing – original draft, Visualization, Validation, Supervision, Software, Resources, Project administration, Methodology, Investigation, Funding acquisition, Formal analysis, Data curation, Conceptualization. **Randy Shively:** Writing – review & editing, Supervision, Investigation, Formal analysis, Conceptualization.

## Data availability

The data used to support our findings are confidential, as required by our various research agreements. In balancing the need for ethics and transparency in our reporting, we provide only aggregated data and further inquiries may be directed to the principle investigator, Rosemary Ricciardelli.

## Funding

This work was commissioned by the Union of Safety and Justice Employees (USJE).

## Declaration of competing interest

The authors declare that they have no known competing financial interests or personal relationships that could have appeared to influence the work reported in this paper.

## Figures and Tables

**Table 1:** Participants (n = 30) by identification number, workplace location, and gender

PIN#	Workplace	Gender
2	Institution	Male
6	Institution	Female
10	Institution	Female
11	Institution	Female
12	Institution	Female
13	Community	Female
14	Community	Female
18	Community	Female
20	Institution	Female
23	Institution	Female
24	Community	Female
27	Community	Female
39	Community	Male
54	Community	Female
57	Community	Female
64	Community	Male
97	Institution	Female
107	Community	Female
109	Institution	Female
112	Institution	Male
117	Institution	Female
119	Institution	Female
130	Community	Female
131	Institution	Female
132	Community	Female
135	Institution	Female
139	Institution	Female
141	Institution	Female
146	Institution	Female
147	Institution	no response

**Table 1.1:** Breakdown of institutional versus community Parole Officers

ROLE	n=	%
CPO	12	40
IPO	18	60
Total	30	100

**Table 1.2:** Breakdown of participants' self-identifying gender

GENDER	n=	%
Female	25	83.33333
Male	4	13.33333
n/a	1	3.333333
Total	30	100 %

**Table 1.3:** Breakdown of participants' age range

AGE	n=
25–34	3
35–44	17
45–54	7
55–64	2
Total	30
